# Why students feel competent in the classroom: A qualitative content analysis of students’ views

**DOI:** 10.3389/fpsyg.2022.928801

**Published:** 2022-10-13

**Authors:** Nadia Catherine Reymond, Ruth Gerlinde Nahrgang, Nadine Großmann, Matthias Wilde, Stefan Fries

**Affiliations:** ^1^Department of Psychology, Faculty of Psychology and Sports Science, Bielefeld University, Bielefeld, Germany; ^2^Department for Didactics of Biology, Faculty of Biology, Bielefeld University, Bielefeld, Germany

**Keywords:** competence satisfaction, need support, mastery goal structure, perceived error climate, reference norm orientation, teaching quality

## Abstract

This qualitative study aimed to identify and to systematize factors that contribute to students’ competence satisfaction in class from students’ perspectives. Based on self-determination theory as our primary theoretical background, we conducted episodic interviews with 25 high school students. A combined deductive-inductive qualitative content analysis approach was applied. As our key finding, we revealed different teaching factors within and beyond self-determination theory (i.e., structure, autonomy support, relatedness support, mastery goal structure, perceived error climate, teaching quality, teachers’ reference norm orientations) as well as additional factors (e.g., students’ motivation and engagement, peer climate and reciprocal peer support) that contributed to students’ competence satisfaction in class from the students’ points of view. This study contributes to existing research on why students’ competence satisfaction arises in class by complementing it with an integrative, explorative, and student-oriented perspective.

## Introduction

Students’ competence satisfaction plays a crucial role for motivation, achievement, and individual growth (Ryan and Deci, 2017; Vansteenkiste et al., 2020; Vasconcellos et al., 2020). Therefore, in the literature, researchers have linked several teaching practices to students’ competence satisfaction (e.g., perceptions of structure and autonomy support) that can be addressed to support students’ competence satisfaction in different educational settings (e.g., school, extracurricular learning; Jang et al., 2010; Guay et al., 2017; Eckes et al., 2018; Aelterman et al., 2019; Ryan and Deci, 2020; Vasconcellos et al., 2020). However, in the context of self-determination theory (SDT), studies investigating the factors that contribute to students’ competence satisfaction have, in part, provided controversial findings. For instance, structure has been beneficial for students’ competence satisfaction when provided in an autonomy-supportive way (Eckes et al., 2018). Autonomy support has, however, partly been negatively correlated with individuals’ competence satisfaction (Steingut et al., 2017; Vasconcellos et al., 2020). Moreover, little is known about students’ views on why their competence satisfaction evolves in class. One reason is that students’ need satisfaction has rarely been studied qualitatively (Hassandra et al., 2003). However, qualitative studies are an important step in order to understand the development and the manifestation of subjective experiences in social contexts through specific perspectives (Ryan and Deci, 2020; Vansteenkiste et al., 2020). To widen researchers’ view on why students’ competence satisfaction arises in classroom contexts from the students’ perspectives and to complement the mainly quantitative studies, this qualitative content analysis study explored students’ narratives about which factors contribute to their competence satisfaction in class.

### Students’ competence satisfaction in self-determination theory

In the context of SDT, the basic psychological need theory describes three basic psychological needs, namely the needs for autonomy, relatedness, and competence (Ryan and Deci, 2017, 2020). The *need for autonomy* is the need to regulate one’s experiences and actions in a self-determined way. The *need for relatedness* is defined as the need to feel socially connected with others. The *need for competence* is the need on which we focus in this study. It is defined as the individuals’ need to experience effectiveness in interactions with their environment (Deci and Ryan, 2000; Ryan and Deci, 2017, 2020). Students’ need for competence is satisfied when students act in and experience classroom environments in which they can express and extend their skills and knowledge (Ryan and Deci, 2020; Vansteenkiste et al., 2020). Moreover, students feel competent when their abilities are in balance with the demands of actions (Reeve, 2015). In the following, the satisfaction of students’ need for competence is referred to as students’ competence satisfaction.

Students’ competence satisfaction is essential for their motivation, achievement, and well-being (Reis et al., 2000; Jeno et al., 2018; Muenks et al., 2018; Ryan and Deci, 2020; Vasconcellos et al., 2020). Contrarily, the frustration of students’ need for competence has been linked to disengagement, amotivation, and helplessness (Legault et al., 2006; Earl et al., 2017; Ryan and Deci, 2017, 2020; for an overview, see Vansteenkiste et al., 2020). These findings show the importance of taking students’ need for competence into account when designing lessons and school environments.

#### Fostering students’ competence satisfaction in self-determination theory

Within SDT, the measures designed to fulfill students’ basic psychological needs are subsumed under the term need support. Need support typically encompasses structure, autonomy support, and relatedness support (Ryan and Deci, 2020). *Structure* describes to which extent teaching styles provide clear communication of expectations, appropriate feedback, and guidance (Jang et al., 2010; Ryan and Deci, 2017, 2020; Aelterman et al., 2019; Vasconcellos et al., 2020). It can be divided into clarifying and guiding structure (Aelterman et al., 2019). Teachers with a focus on clarifying structure give overviews about the learning material, make their expectations transparent, and monitor the students’ progress. Teachers who apply guiding structure provide help and guidance when needed. They also assist the students to accept mistakes as an important step in their learning progress, as well as to reflect on them (Aelterman et al., 2019).

*Autonomy support* focuses on identifying and nurturing students’ feelings, perspectives, and preferences (Jang et al., 2010; Ryan and Deci, 2017, 2020; Aelterman et al., 2019; Vasconcellos et al., 2020). It has been divided into participative and attuning autonomy support (Aelterman et al., 2019). Teachers focusing on participative autonomy support engage in dialogue with their students. They invite them to provide input and give them opportunities to choose. Attuning autonomy support comprises the acceptance of students’ feelings, the provision of meaningful rationales, and the application of ways to make learning enjoyable for the students (Aelterman et al., 2019).

*Relatedness support* includes teaching practices that empower students’ sense of social connection and belonging (Reeve, 2015; Sparks et al., 2016; Vasconcellos et al., 2020). The latter has scarcely been explored in SDT (Sparks et al., 2016). However, following physical education research, relatedness-supportive teachers provide individualized conversations, task-related feedback, and promote cooperation and teamwork. They also show enthusiasm, have high awareness, care about their students, and communicate in a friendly way with them (Sparks et al., 2015, 2016). Reeve (2015) has additionally proposed relatedness support to comprise the following aspects: taking time for other individuals, caring and knowing things about other individuals, expressing affection and appreciation with regard to other individuals, enjoying interaction, and sharing resources (e.g., interest) with other individuals.

From an empirical point of view, autonomy support and structure have been positively associated with students’ competence satisfaction quite consistently (Patall et al., 2008; Mouratidis et al., 2013; Guay et al., 2017; Ryan and Deci, 2020; Vasconcellos et al., 2020). For instance, meta-analytical findings have shown a strong link between structure and students’ competence satisfaction as well as a positive relationship between opportunities to choose and students’ competence satisfaction (Patall et al., 2008; Vasconcellos et al., 2020). Furthermore, relatedness support was positively correlated to students’ competence satisfaction in a meta-analysis (Vasconcellos et al., 2020). Relatedness was also a major theme for youth in a social service context (Nagpaul and Chen, 2019). These findings suggest that relatedness support could play an important role for students’ perspectives on which factors contribute to their competence satisfaction.

Still, first, compared to autonomy-supportive measures, SDT research has paid less attention to measures that foster students’ competence satisfaction (Sparks et al., 2016; Vasconcellos et al., 2020). Second, these findings have partly been controversial (Guay et al., 2016; Steingut et al., 2017; Vasconcellos et al., 2020). This controversy impedes implications on why students feel competent in class. It prompts more research on which factors contribute to their competence satisfaction. Third, the typically applied approach describing teachers’ need support does not make claims about completeness (Vansteenkiste et al., 2020). Factors within and especially factors that go beyond perceptions of teaching practices, such as student factors, peer factors, and context factors, remain to be explored. Last, there is a lack of studies that explore students’ perspectives on how and why need-supportive measures influence their competence satisfaction in class (Anderman et al., 2002; Ryan and Deci, 2020). However, students are one of the actors in classes as social contexts. Their perspectives are hence important in order to understand the motivational processes taking place within and across classrooms (Nolen et al., 2015; Nolen, 2020).

### Understanding why students feel competent in class – The need for qualitative and integrative research

Qualitative research is able to provide a deep understanding of students’ narratives and experiences, to describe even complex student-environment-interactions, and to reveal how and why need-supportive measures work through individuals’ perspectives (Patrick et al., 2001; Anderman et al., 2002; Flick, 2011; Nolen et al., 2012, 2015; Mayring, 2014; Nolen, 2020; Ryan and Deci, 2020; Vansteenkiste et al., 2020). Additionally, qualitative studies enable researchers to take a holistic perspective (Nolen et al., 2015; Vansteenkiste et al., 2020). That is because, for instance, qualitative studies can simultaneously consider a theory-based perspective (i.e., deductive thinking; the use of existing theory in deriving qualitative findings) and a data-based perspective (i.e., inductive thinking; the explorative analysis of data; Mayring, 2014). Furthermore, qualitative research facilitates the transfer of theoretical knowledge into school practice, because it offers more detailed insights into individuals’ behaviors and experiences compared to quantitative research (Patrick et al., 2001; Mayring, 2014; Nolen et al., 2015; Ryan and Deci, 2020; Vansteenkiste et al., 2020). Qualitative research is hence one useful approach to widen researchers’ view on which factors contribute to students’ competence satisfaction through students’ perspectives. In line with this, Ryan and Deci (2020) have called for more qualitative research in the context of need support.

Moreover, scientific knowledge is primarily gained by building on existing research (Merton, 1957; Parolo et al., 2020). Accordingly, researchers in motivational psychology as well as in methodological literature have called for combining and integrating different theoretical frameworks in order to extend, refine, and integrate existing knowledge (Mayring, 2016; Anderman, 2020; Flick, 2020). Such an integrative approach is particularly important when aiming to translate specific research questions (e.g., why students’ competence satisfaction arises in class) into comprehensible recommendations for practitioners in the classrooms (e.g., teachers; Anderman, 2020). After having reached several findings and contributions, translations into practitioner-oriented recommendations have been called for in the context of SDT (Ryan and Deci, 2020). Therefore, one important question is which existing theories one can build on in addition to SDT. Besides taking the students’ perspectives into account, this work took an integrative perspective, and considered different theoretical frameworks in order to widen SDT researchers’ view on how to facilitate students’ competence satisfaction in class.

### Understanding why students feel competent in class – Theoretical frameworks for qualitative research

The investigation of teaching practices is one approach which has extensively been investigated in motivational and educational psychology (Lazowski and Hulleman, 2016; Ryan and Deci, 2020; Vansteenkiste et al., 2020). Based on a continuous dialogue with experts in motivational psychology, didactics, and educational psychology, as well as on a literature informed strategy that sought to include renowned works (e.g., Corno and Anderman, 2016; Wentzel and Miele, 2016), we therefore preselected a variety of teaching practices that might contribute to students’ competence satisfaction beyond existing SDT assumptions from students’ perspectives. Specifically, some well-established teaching practices have been essential for educational outcomes, and have already been linked to students’ competence satisfaction or to related perceptions of competence (e.g., Halvari et al., 2011; Steuer et al., 2013; Scherer et al., 2016; Dickhäuser et al., 2017). In order to identify and to systematize additional factors that might contribute to students’ competence satisfaction beyond the existing SDT assumptions from our integrative perspective, we therefore considered the theoretical frameworks from which these teaching practices were derived, namely the achievement goal theory, perceived error climate, teaching quality, and reference norm orientation theory, in the conceptualization, analysis, and discussion of the present study. They are outlined hereafter.

In the *classroom goal structure* literature, researchers typically distinguish between *mastery goal structure* (a focus on developing competencies in class), *performance approach goal structure* (a focus on demonstrating competence and on outperforming others in class), and *performance avoidance goal structure* (a class focus on not demonstrating incompetence and on avoiding to be inferior to others in terms of performance; Meece et al., 2006; Urdan and Schoenfelder, 2006; Schwinger and Stiensmeier-Pelster, 2011). Classroom goal structures have been an important starting point for motivational interventions as well as for understanding students’ motivational and achievement-related functioning (e.g., Wolters, 2004; Urdan and Kaplan, 2020). Moreover, having a high level of mastery goal structure has been positively linked to perceptions of competence satisfaction (e.g., Kavussanu and Roberts, 1996; Cox and Williams, 2008; Halvari et al., 2011). Taking the well-investigated TARGET approach into account (Ames, 1992; Meece et al., 2006; Lüftenegger et al., 2014; Urdan and Kaplan, 2020), the following mastery goal structure dimensions could thus help to investigate students’ perspectives on why their competence satisfaction arises in class: task (teachers design tasks that focus on learning, provide optimal challenge, and enable students’ active involvement), authority (teachers provide opportunities to choose, for sharing perspectives, and for taking responsibility), recognition (teachers recognize students’ acting and achievement, e.g., by using feedback), grouping (teachers enable collaborative work in heterogeneous groups and interaction among students), evaluation (teachers’ evaluations focus on learning and collaboration instead of competition), and time (teachers provide appropriate workload and pace; Meece et al., 2006; Lüftenegger et al., 2014, 2017).

Another theoretical approach which we addressed is the *perceived error climate* research (e.g., Oser and Spychiger, 2005; Steuer et al., 2013; Reeve, 2015). Perceived error climate is defined as the way of evaluating and using errors within learning processes in classroom environments or other social learning environments (Steuer et al., 2013). With regard to classroom contexts, Steuer et al. (2013) described the perceived error climate as a multidimensional construct including eight dimensions such as teachers’ error tolerance. Perceived error climate has not yet been linked to students’ competence satisfaction but to students’ self-concept and employees’ self-efficacy as competence-related variables (Putz et al., 2013; Steuer et al., 2013). It also partly appeared in the literature on need-supportive measures (Reeve, 2015; Aelterman et al., 2019; Jiang et al., 2019). These theoretical and empirical discussions suggest that a positive error climate might help to identify additional factors that contribute to students’ competence satisfaction through students’ perspectives.

In the *teaching quality* framework, researchers typically define three basic dimensions in order to explain under which circumstances students can learn effectively: *classroom management* (getting and keeping students attentive and on task), *cognitive activation* (providing optimal challenge and fostering students’ thinking), and *student support* (establishing a teacher–student-relationship which fulfills students’ needs; Praetorius et al., 2018). The teaching quality dimensions are one main precondition for self-perceptions of competence related to students’ competence satisfaction (e.g., self-concept) as well as for students’ achievement which again is related to students’ competence satisfaction (Weinert et al., 1989; Scherer et al., 2016; Jeno et al., 2018; Praetorius et al., 2018; Blömeke and Olsen, 2019). Moreover, the student support dimension has been elaborated based on the need-supportive measures (Praetorius et al., 2018). Hence, first empirical findings and theoretical elaborations indicate the relevance of teaching quality for students’ competence satisfaction. In contrast to this and the importance of this framework for several educational processes (e.g., Fauth et al., 2014; Scherer et al., 2016; Panayiotou et al., 2021), the teaching quality dimensions have not been empirically linked to students’ competence satisfaction in terms of SDT.

In *reference norm orientation* theory (e.g., Rheinberg, 1980, 1983; Dickhäuser et al., 2017), researchers distinguish between three reference norms: The *social* (the use of interindividual comparisons), *criteria-oriented* (the use of comparisons with an absolute standard), and *intraindividual reference norm* (comparing students’ achievement with their own prior achievement) describe comparison standards by which actions, performance or competence are evaluated (Rheinberg, 1980, 1983; Dickhäuser et al., 2017; Lohbeck and Freund, 2021). Teachers use some reference norms more frequently than others which is called teachers’ reference norm orientation. Specifically, teachers who are oriented toward the intraindividual reference norm focus on improvement, have short-term expectations, and provide optimal challenge, among others. Teachers who are oriented toward the social reference norm focus on normative competence and provide uniform tasks for all students in class. Teachers who frequently use criteria-oriented reference norms presumably apply criteria-oriented teaching and task-focused feedback (Rheinberg, 1980, 1983; Dickhäuser et al., 2017; Lohbeck and Freund, 2021). However, teachers’ criteria-oriented reference norm orientation has not been elaborated yet. With regard to students’ competence satisfaction, teachers’ intraindividual reference norm orientation and teachers’ use of the criteria-oriented reference norm were found to be positively associated with related self-perceptions of competence (e.g., self-concept; Rheinberg, 1983; Krampen, 1987; Lüdtke et al., 2005; Dickhäuser et al., 2017; Lohbeck and Freund, 2021). Furthermore, in the context of SDT, some theoretical considerations as well as initial findings stressed the importance of differentiated instruction and improvement-focused feedback which are key elements of teachers’ intraindividual reference norm orientation (Carpentier and Mageau, 2013; Reeve, 2015; Guay et al., 2017; Ryan and Deci, 2017). Although this prompts further research on whether teachers’ reference norm orientations might contribute to students’ competence satisfaction in terms of SDT, studies have not addressed this linkage.

To conclude, more research is required with respect to the competence-supportive measures within SDT, given the mainly quantitative, and some controversial findings in past research. Specifically, more qualitative research on students’ perspectives is required in order to take their essential perspectives into account in realistic classroom contexts. Furthermore, first hints suggest that, besides SDT and students’ perspectives, the depicted additional theoretical frameworks (i.e., the research on classroom goal structures, perceived error climate, teaching quality, and reference norm orientations) could provide additional factors that contribute to students’ competence satisfaction in the sense of SDT (e.g., Halvari et al., 2011; Dickhäuser et al., 2017; Praetorius et al., 2018). However, those frameworks as well as factors going beyond teaching practices (e.g., student factors, peer factors, and situational factors) have not sufficiently been considered with regard to students’ competence satisfaction in the context of SDT. A combined explorative investigation of students’ perspectives and integrative consideration of the depicted theoretical backgrounds hence is one fruitful approach to extend the existing literature on which factors contribute to students’ competence satisfaction through students’ perspectives.

Due to its procedure variety which allows a combined theory-driven and data-driven perspective, the qualitative content analysis is one approach which is particularly suitable in addressing these research desiderata by using qualitative material (Mayring, 2014). Moreover, its rule-oriented as well as hybrid (i.e., combined qualitative and quantitative) or, in other words, integrated (i.e., combination of qualitative and quantitative analysis steps within one research design) approach allows an exact elaboration, validation, and further analysis of qualitative categories (Mayring, 2007a,^2014^; Burzan, 2016; Gläser-Zikuda et al., 2020). It is noteworthy that the need support, mastery goal structure, perceived error climate, teaching quality, and the reference norm orientation frameworks partly encompass similar teaching practices (e.g., optimal challenge; Rheinberg, 1983; Lüftenegger et al., 2017; Praetorius et al., 2018; Aelterman et al., 2019). From a pragmatical perspective, a combined theory- and data-based as well as a hybrid (or: integrated) approach to analyze qualitative material therefore seems particularly promising in order to get a holistic view of separable factors which represent reasons for students to feel competent in the classroom (Mayring and Brunner, 2006; Mayring, 2007a,^2014^; Burzan, 2016). By applying a combined theory-based and data-based content-analytical approach, one may hence identify and systematize already known (e.g., structure) and additional factors (e.g., student factors) that contribute to students’ competence satisfaction in class from the students’ perspectives.

### Present study

Based on existing research, this interview study aimed to identify and to systematize additional factors that might contribute to students’ competence satisfaction. In addition to SDT, it focused on students’ perspectives (i.e., explorative research design; Mayring, 2007a,^2014^) as well as on existing theoretical frameworks (classroom goal structure literature, perceived error climate research, teaching quality framework, reference norm orientation theory) that might add to SDT with regard to students’ competence satisfaction in class (i.e., descriptive research design; Mayring, 2007a,^2014^). As a result, this qualitative study had a combined explorative-descriptive field research design. By doing so, it aimed to refine, extend, and integrate existing knowledge within and beyond SDT on how to fulfill students’ need for competence in realistic classroom settings, giving new directions for future research. By using episodic interviewing, this work addressed students’ generalized beliefs as well as the complexity of student-classroom environment-interactions in real school-life situations (Flick, 2011, 2018). By analyzing the interviews following the rules of qualitative content analysis, this study applied a rigorous and hybrid (or: integrated; i.e., combined qualitative and quantitative) approach for analyzing qualitative material (Mayring, 2000, 2007a,^2014^; Mayring and Brunner, 2006; Scheufele, 2008; Burzan, 2016; Krippendorff, 2019; Gläser-Zikuda et al., 2020; e.g., Duchatelet et al., 2020). The research question that we addressed in our qualitative, integrative, and student-oriented study was: Which factors contribute to students’ competence satisfaction through students’ perspectives? The study was cross-sectional in nature, focusing the representativeness of our sample (Nolen et al., 2012).

## Materials and method

### Participants and procedure

The present study took place from May to July, 2019. It involved *N* = 25 ninth-grade students (*n* = 9 male, *n* = 16 female) from two high schools (in German: Gymnasium) in the state of North Rhine-Westphalia, Germany. The students’ mean age was 14.84 years (*SD* = 0.47 years). For the purpose of sampling, we deliberately addressed two contrasting schools in order to represent the perspectives of students’ coming from schools as diverse as possible, namely a private school in a rather rural area with a rather low socioeconomic status and a public school in a large city (Küpper, 2016; Landatlas, 2019). This contrast-oriented sampling procedure is common in qualitative and mixed-methods research in order to overcome biased material (e.g., due to over-representing specific contexts) and to enhance validity (Krippendorff, 1989; Brink, 1993; Collins et al., 2007; Onwuegbuzie and Leech, 2007; Creswell and Poth, 2016; e.g., Flick et al., 2019). After having obtained consent from the principals and teachers within the addressed schools, we orally presented the study, distributed information material as well as the written informed consent forms, and asked the students for participation during school lessons. This procedure combines the depicted purposive sampling procedure with a convenience sampling procedure. By doing so, we warranted the availability and willingness of the individual students to participate (Collins et al., 2007). According to the clarity of the research field, the expected data quality, as well as the expected heterogeneity of participants, the first 25 students (School 1: *n* = 15; School 2: *n* = 10) who were willing to participate were included in the study (Guest et al., 2006). *Post hoc* analyses revealed that data saturation was reached after coding 51% of the interview material.

According to the European Union General Data Protection Regulation 2016/679 and the Data Protection Act of North Rhine-Westphalia (Germany), all participants were informed about the voluntary nature of participation, and gave their written informed consent before the beginning of the interview. For students who were younger than 16 years old at the time of the interview, written parental consent was additionally provided. According to the depicted regulations, the written informed consent included content information (e.g., information on the study aims and procedures), legal information (e.g., the right to withdraw from the study), and the declaration of consent itself. The study was approved by the responsible research ethics committee.

In order to standardize the interview procedure, we conducted semi-structured episodic interviews according to Flick (2011) (*M*_*duration*_ = 42.86 min; *SD*_*duration*_ = 12.87). Episodic interviews are a combination of narrative and semi-structured interviews. They contain open-ended questions and situation-specific narratives in order to capture both episodic and semantic components of students’ subjective narratives and experiences (Flick, 2018). The applied interview schedule (see Supplementary Appendix A) was revised after one pilot interview. In accordance to this interview schedule, the interviewers first introduced the topic of the study as well as the procedure and then asked the students to define their understanding of the term “competence satisfaction.” Second, the interviewers defined the aforementioned term in the sense of SDT. If necessary, the interviewers gave a standardized example. Third, the interviewers and students settled on a common definition according to the SDT definition. Fourth, the interviewers asked the students to describe at least one situation in which the students had perceived competence satisfaction in class, and at least one situation in which the students had perceived competence frustration (e.g., “Can you remember a current classroom situation [from the ongoing school year] in which you felt particularly competent? Please tell me about this situation.”). Fifth, the interviewers explored the students’ general beliefs about and experiences with factors that contribute to their competence satisfaction and with factors that contribute to their competence frustration in class. For this purpose, they asked some questions about the general reasons and circumstances under which the students felt competent and incompetent in class (e.g., “In general, what helps you in class to feel competent or what is important for you in class so that you can feel competent?”). Based on our research question, the situations in which the students perceived competence frustration and the generalized beliefs about and experiences with competence frustration were not of further relevance within this work. Last, the interviewers and students completed a short demographics questionnaire together.

The interviews were audio-recorded and conducted in one-to-one-settings (one interviewer, one student). They took place in a private room of the respective schools. In order to reach an adequate level of closeness and distance between interviewers and interviewees (Helfferich, 2011), the interviewers were two student teachers. Given that the interviewers both had a more similar age and background to the interviewees than the authors of this study, it was assumable that the students would open up more easily by doing so. Nevertheless, the interviewers had sufficiently divergent backgrounds from the interviewees to uphold the interviewees’ willingness to verbalize information that is obvious to insiders but necessary to interpret the interview data (Helfferich, 2011).

As far as professionality is concerned (Helfferich, 2011), the interviewers had a strong school background (recent school experiences; a Bachelor’s degree in teaching, a well-advanced Master’s program in teaching), as well a motivational psychology background (successfully accomplished courses in motivational psychology). In addition, the interviewers were trained by the corresponding author of this study before the survey began. The training included the working through the literature which underlay our interview approach along with its debriefing (Flick, 2011, 2018; Helfferich, 2011), the discussion of the interview schedule and of questions, as well as the practicing of the interviews among the interviewers and in the mentioned pilot interview. The practicing interview and the pilot interview were debriefed with the corresponding author (practicing interview, pilot interview) and the pilot interviewee (pilot interview). The interview process and the Master thesis projects in the context of which the student teachers collected the data of the present study was supervised by the corresponding author (i.e., psychologist; researcher in the fields of motivational psychology and educational psychology), the third author (Master’s degree in teaching; researcher in the fields of biology didactics and motivational psychology), and the fourth author (i.e., teacher; researcher in the fields of biology didactics and motivational psychology) of the present study.

Beyond their important roles within the data collection of this study (investigation), the student teachers supported the participants’ acquisition (resources). The corresponding author provided the study materials (investigation), and took responsibility of the conceptualization, data curation, visualization, methodology, the writing of the original draft, and the project administration. In addition, the corresponding author and the second author of this manuscript (i.e., psychologist; researcher in the fields of motivational psychology and educational psychology) formally analyzed the interview material. A continuous peer review and peer debriefing across the author team was established during the entire research process, e.g., during the writing in review and editing stages. The last author of this manuscript (i.e., Master’s degree in Social Science; psychologist; researcher in the fields of motivational psychology, instructional psychology, and educational psychology) provided the resources and supervised the research project (CRediT, 2020).

### Qualitative and quantitative data analysis

**FIGURE 1:**
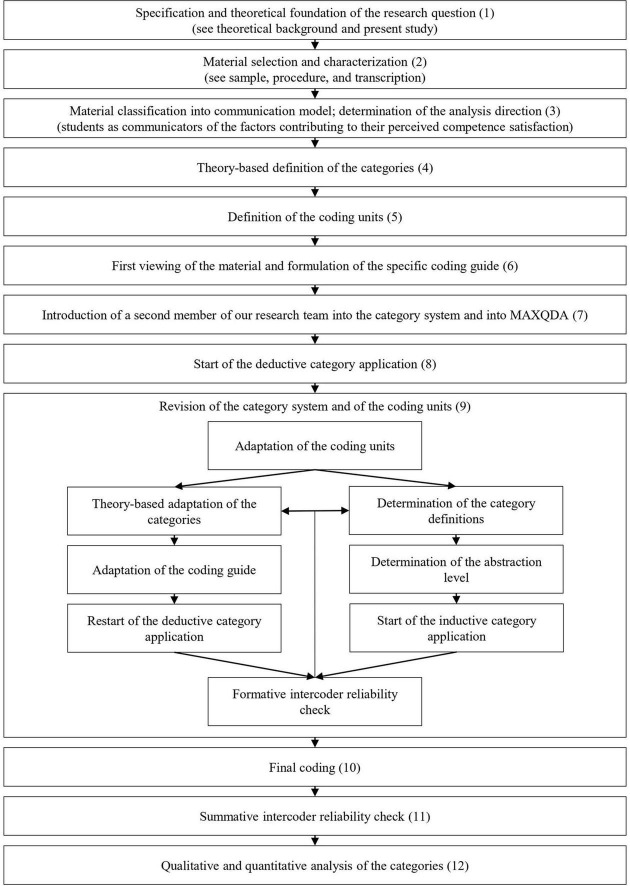
Procedural model of the qualitative content analysis applied in the present study. Adapted from Mayring and Brunner (2006).

After completion of the interviews, we transcribed and anonymized the interviews based on the well-established recommendations of Dresing and Pehl (2018), Kuckartz (2010, 2018), and Selting et al. (2009; see Supplementary Appendix B for the transcription rules). The applied transcription rules represent a verbatim data transcription, except that they slightly adapt spoken language into standard German and to the written language. Moreover, standardized symbols are implemented to highlight specific audio recordings’ characteristics (e.g., […] for one-second-breaks in speaking). In order to anonymize the interviews, we anonymized any names, sites, and assigned a code to each transcript. Afterward, we analyzed the interviews according to qualitative content analysis (Mayring and Brunner, 2006; Schilling, 2006; Mayring, 2014; Krippendorff, 2019). Qualitative content analysis is a hybrid (or: integrated) analysis approach that combines a rigorous qualitative and quantitative analysis of qualitative communication material, such as text material (Mayring, 2007a; Burzan, 2016; Gläser-Zikuda et al., 2020). Its qualitative analysis steps represent a phenomenological description of the interview material that is narrowly based on the interviewees’ statements. They result in a category system which gives a structured overview about the contents of the specific communication material with regard to a specific research question (Mayring, 2014). Subsequent quantitative analysis steps regarding the resulting category system enable an exploration and description of the salience of specific categories within investigated samples, among other possibilities (Mayring, 2014). An overview about the procedural model applied in our study can be found in Figure 1. Note that Steps 1 to 3 in Figure 1 have already been considered in the sections theoretical background, present study, sample, procedure, and in the description of our transcription procedure.

Since we combined the deductive (i.e., theory-based derivation of categories) and inductive (i.e., material-based derivation of categories) approach of qualitative content analysis in this study (Mayring and Brunner, 2006; Schilling, 2006; Mayring, 2014), we first elaborated the categories and definitions for the deductive category system (Step 4 in Figure 1). We thereby relied on the aforementioned theoretical frameworks (i.e., SDT, classroom goal structure literature, perceived error climate research, teaching quality framework, reference norm orientation theory) and on our interview schedule.

Then, we segmented the interview material into 8087 coding units in total (Step 5 in Figure 1; Chi, 1997; Schilling, 2006; for the segmentation rules, see Supplementary Appendix C). Note that the participants are referred to by their codes (e.g., KM01) and by the corresponding interview segment (e.g., 80) to ensure anonymity in any participant quotations (e.g., female student KM01, 15 years old, reported in interview segment 80 “[Teachers who help me feel competent] above all, they explain things well.”). Based on our research question, we identified 1774 coding units describing factors that contributed to students’ competence satisfaction from the students’ perspectives (coding units per interview: *M* = 70.96; *SD* = 30.17). Specifically, we analyzed 34 situations (*n* = 722 segments) in which the students had perceived competence satisfaction. Thousand fifty-two segments contained students’ general beliefs about and experiences with factors that contributed to their competence satisfaction. The remaining coding units referred either to another research question or did not contain any relevant information. They were therefore not analyzed within this work.

With regard to this work, the recording unit was one word, and minimally contained one proposition (i.e., one episode, one idea or one piece of information which is comprehensible by itself) describing a factor that contributed to students’ competence satisfaction. The context unit was one paragraph, and maximally contained one proposition describing one factor that contributed to students’ competence satisfaction (Schilling, 2006; Tesch, 2013). The unit of classification was all coding units out of one interview referring to factors that contributed to students’ competence satisfaction since we chose a cross-interview approach (Schilling, 2006; Mayring, 2014).

After an initial viewing of the material, provisional coding rules and text examples were inserted into the deductive category system (Step 6 in Figure 1). A second member of our research team was introduced to the category system and to the applied analysis software MAXQDA 2020 (Step 7 in Figure 1; VERBI Software, 2019). Afterward, both main coders commonly coded 10% of the material in order to get familiar with the category system, and to identify initial ambiguities in the category system (Step 8 in Figure 1). After solving those ambiguities (Step 9 in Figure 1), both main coders independently coded the same 24% of the interview material while documenting difficult coding units and categories. After a subsequent formative intercoder reliability check, further problems in the category system were discussed, identified, and resolved (Schilling, 2006; Mayring, 2014). Specifically, minor overlaps between different categories or minor ambiguities entailed the addition or revision of definitions, coding rules, and text examples. For instance, we added coding rules that stressed the difference between *Clear communication and high-quality explanations* (coding units generally referring to adequate explanations when no support is required), *Optimal challenge for student and regarding school requirements* (coding units specifically describing teacher explanations having an appropriate challenge level regarding the students’ stage in learning progresses or regarding school requirements when no support is required), and *Constructive and appropriately challenging support* (coding units describing the usefulness of additional explanations the teachers use to support the students when support is required). Larger overlaps between categories entailed the integration of multiple categories if reasonable. For instance, we combined *Participation possibilities* and *Autonomy-supportive interaction* (see *Participation possibilities and autonomy-supportive interaction)* which were originally placed in two separate categories. Supplemental categories or subcategories were inductively added if both raters agreed about the fulfillment of the following criteria: (a) the content did not fit into the existing categories, (b) the interviewed students viewed this content as a factor that contributed to their competence satisfaction, (c) the content arose several times, (d) the integration of this content into existing categories would have biased the original categories. One deductive category (*Meta-cognition support*; Praetorius et al., 2018) was removed because it did not arise in our sample. Based on Mayring (2016), we also adapted the coding units retroactively in order to calibrate the coding units to the applied abstraction level of analysis. In line with the iterative character of qualitative content analysis, the analysis steps in Step 9 of Figure 1 were each repeated by coding a further 10% of the interview material until formative intercoder reliability was acceptable (Mayring and Brunner, 2006; Schilling, 2006; Mayring, 2014; Krippendorff, 2019).

For the final coding (Step 10 in Figure 1), the 25 interviews were randomly assigned to the two main coders (main coder 1: *n* = 15 interviews; main coder 2: *n* = 14 interviews; note that *n* = 4 of the *N* = 25 interviews were coded twice in order to perform the summative intercoder reliability check). In order to ensure that the final category system^1^ works with interchangeable coders, a third coder (i.e., student assistant in the research field of motivational psychology and educational psychology; combined Bachelor’s degree in linguistics and psychology; advanced double degree in linguistics [Master program] and psychology [Bachelor program]) was involved in the summative intercoder reliability check after a short briefing regarding the final category system (Step 11 in Figure 1; Mayring and Brunner, 2006; Hayes and Krippendorff, 2007; Mayring, 2014). Krippendorff’s alpha (α = 0.74; 95% CI [0.71–0.77]) indicated an acceptable intercoder reliability (Krippendorff, 2019).

We subsequently conducted qualitative and quantitative analyses of the interview material (Step 12 in Figure 1; Mayring and Brunner, 2006; Schilling, 2006; Mayring, 2014). In the course of the quantitative analyses, we conducted three indicators in order to identify the salience of the categories and subcategories within our sample. According to Schilling (2006), we analyzed the absolute topic frequencies, which are the absolute frequency of coding for each subcategory across all students. Second, we analyzed how many students had addressed each subcategory in at least one segment, and at least from the perspective of one coder (i.e., person frequency; Schilling, 2006). Third, we analyzed the relative distribution of the different subcategories based on the person frequencies.

As has been implicitly addressed, multiple strategies have been used to establish reliability and validity in the present study: Besides a thick description of the study procedures, a standardized coding system, and an intercoder-reliability check ensured reliability (Hayes and Krippendorff, 2007; Mayring, 2014; Morse, 2015). Regarding validity, triangulation, a continuous peer review, elements of negative case analysis, and the reflection on researcher bias complemented the depicted reliability criteria. Triangulation describes the complementation of multiple investigators, theories, methods, and data with each other to address a research question, and is used to reveal the complexity of investigated phenomena (Mayring, 2007a; Morse, 2015; Flick et al., 2019). A data triangulation took place, since we interviewed interviewees’ visiting two contrasting schools, conducted episodic interviews which explore both interviewees’ past experiences and current concepts, and quantitatively assessed the demographics (Mayring, 2007a; Flick et al., 2019). An investigator triangulation was given, since multiple stakeholders contributed different perspectives to the present study, for instance, in form of the intercoder-agreement-check (Mayring, 2007a; Krippendorff, 2019). A theory triangulation was applied, because we confronted the data with the theoretical backgrounds of self-determination theory, mastery goal structure, perceived error climate, teaching quality, and reference norm orientation (Mayring, 2007a). A methodological triangulation was part of the study, since the qualitative content analysis represents a hybrid (i.e., combined qualitative and quantitative) or integrated (i.e., a combination of qualitative and quantitative analysis steps in one research design) analysis of qualitative material which additionally combines explorative (inductive) and hypothesis-oriented (deductive) analysis procedures (Mayring and Brunner, 2006; Mayring, 2007a; Burzan, 2016). Concerning negative cases, the coders were attentive to categories that were salient (i.e., positive cases) and non-salient (i.e., negative cases) through the interviewed students’ perspectives (Morse, 2015). Member checks were not applied in this study because they are not unconditionally recommended in interview research (e.g., Morse, 2015). Moreover, there are no clear recommendations on how to deal with potential differences between the researchers’ judgments and the participants’ judgments (Morse, 2015).

## Findings

On average, the students each described 1.36 situations in which they had perceived competence satisfaction (*SD* = 0.57; *Min* = 1; *Max* = 3). The situations in which the students perceived competence satisfaction most commonly arose in the school subjects Mathematics (29%), History (15%), and English (12%). The students also reported situations out of eight further school subjects in which they had perceived competence satisfaction. On average, students’ current school grades in the reported school subjects were *M* = 1.85 (*SD* = 0.94; *Min* = 1; *Max* = 4). In Germany, school grades range from 1 (*very good*) to 6 (*unsatisfactory*).

**FIGURE 2:**
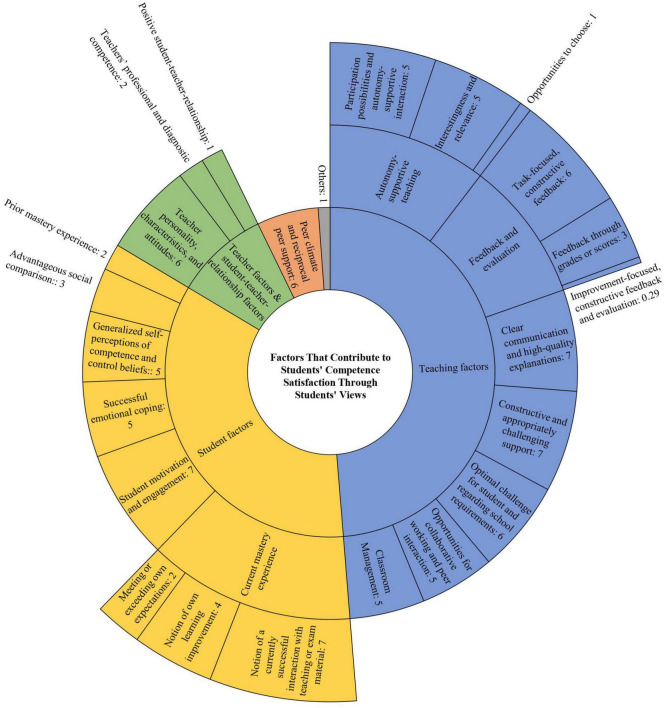
Relative category distribution of the factors that contribute to students’ competence satisfaction through students’ perspectives. The relative category distribution represents *n*% of all category codings, based on the absolute person frequencies (i.e., *n* students that have addressed the respective categories at least in one segment and at least from the perspective of one coder; see Table 1). The relative category distributions sum up to 101.29% instead of 100% because, for the sake of clarity, all values have been rounded to the nearest whole number, with one exception. The relative distribution of the category “Improvement-focused, constructive feedback and evaluation” has been rounded to two decimal places in order not to create the impression that its value is 0%.

Table 1 and Figure 2 provide overviews of the final category system describing the factors that contributed to students’ competence satisfaction in class through the students’ perspectives both for the situations and the generalized beliefs and experiences (including the category labels and quantitative analyses). Specifically, Table 1 shows the categories, the absolute topic frequencies, and the relative person frequencies for the categories. Figure 2 illustrates the categories and the relative distributions of the categories (for the three frequency types, see the quantitative frequency indicators in the method section). As a finding, the category system comprised five main categories (e.g., *Teaching factors*; including *Others*), subsuming 16 categories (e.g., *Constructive and appropriately challenging support*), that, in turn, comprised nine subcategories (e.g., *Task-focused, constructive feedback*). In the following, we present our findings along the main category sequence *Teaching factors*, *Teacher factors and student-teacher relationship factors*, *Student factors*, and *Peer climate and reciprocal peer support* as displayed in Table 1. Within those, we focused on the subcategories and categories with a person frequency higher than 50% and on surprising findings due to limited space. By doing so, we attempted to report the findings that were representative for large amounts of our sample or that gave new directions for future research (Schilling, 2006).

**TABLE 1:** Factors contributing to students’ competence satisfaction through students’ perspective.

Categories	Frequencies	
Main categories Categories Subcategories	Absolute topic frequency^a,b^	Relative person frequency^c^
Teaching factors (T)		
Constructive and appropriately challenging support (T1)	235	92
Clear communication and high-quality explanations (T2)	64	92
Optimal challenge for student and regarding school requirements (T3)	122	84
Feedback and evaluation (T4)		
Task-focused, constructive feedback (T4.1)	102	80
*Feedback through grades or scores (T4.2)*	25	44
Improvement-focused, constructive feedback and evaluation (T4.3)	1	4
Autonomy-supportive teaching (T5)		
Participation possibilities and autonomy-supportive interaction (T5.1)	70	72
Interestingness and relevance (T5.2)	64	64
Opportunities to choose (T5.3)	6	8
Classroom Management (T6)	44	68
Opportunities for collaborative working and peer interaction (T7)	51	68
*Teacher factors and student-teacher-relationship factors* (TR)		
*Teacher personality, characteristics, and attitudes* (TR1)	84	84
*Teachers’ professional and diagnostic competence* (TR2)	12	24
*Positive student-teacher-relationship* (TR3)	30	20
*Student factors* (S)		
*Student motivation and engagement* (S1)	310	100
*Current mastery experience* (S2)		
*Notion of a currently successful interaction with teaching or exam material* (S2.1)	324	100
*Notion of own learning improvement* (S2.2)	21	56
*Meeting or exceeding own expectations* (S2.3)	27	32
*Successful emotional coping* (S3)	29	68
*Generalized self-perceptions of competence and control *beliefs** (S4)	30	64
*Advantageous social comparison* (S5)	57	40
*Prior mastery experience* (S6)	9	24
*Peer climate and reciprocal peer support* (P)	180	84
*Others (O)*	15	16

Deductive categories are written in non-italicized letters. Inductive categories are marked by italicized letters. ^a^Absolute frequency of coding for each category across all students in *n* segments. ^b^Frequencies sum up to *n* = 1912 instead of *n* = 1774 because of multiple coding (*n* = 37), differing coding across raters (*n* = 105), and null coding (*n* = 4). ^c^n% of students that have addressed the respective categories at least in one segment and at least from the perspective of one coder.

### Teaching factors

In line with our initial deductive category system (see the deductive categories, as presented in Table 1), the students reported different *Teaching factors* (T) as factors that contributed to their competence satisfaction in class. This main category included students’ perceptions of teaching styles that contributed to students’ competence satisfaction and could be divided into eight categories (e.g., *Constructive and appropriately challenging support*), with two categories (i.e., *Autonomy-supportive teaching*; *Feedback and evaluation*) being further divided into three subcategories (e.g., *Participation possibilities and autonomy-supportive interaction*; e.g., *Task-focused, constructive feedback*).

Among the *Teaching factors*, *Constructive and appropriately challenging support* as well as *Clear communication and high-quality explanations* were the two most salient (sub-) categories from the views of our participants. Referring to the category *Constructive and appropriately challenging support* (T1), the students described that they felt competent because their teachers helped them at an appropriate challenge level when they required support. For instance, the students reported to feel competent because the teachers repeatedly explained content when something was unclear. Moreover, the students described that they felt competent because teachers created opportunities for asking questions and discussing students’ questions (e.g., female student AT04, 15 years old, reported in interview segment 45 “[A teaching attribute that helps me feel competent is that] if you have questions you can approach the teacher at any time.”). In the category *Clear communication and high-quality explanations* (T2), the students described that they felt competent because of teacher behaviors, such as making expectancies and procedures clear or explaining instructions and the material in an understandable manner. For example, the students frequently stressed that they felt competent because the teachers appropriately explained the learning material or instructions (e.g., female student SD03, 14 years old, reported in interview segment 49 “Simply if the task is well explained, the assignment.”; and female student KM01, 15 years old, stated in interview segment 80 “[Teachers who help me feel competent] above all, they explain things well.”).

A further frequent category was the category *Optimal challenge for student and regarding school requirements* (T3). Within this category, the students described that they felt competent because teachers set appropriate challenge levels for the students but also for the mastering of upcoming school requirements such as exams. Specifically, an appropriate challenge level was defined as neither under- nor overdemanding for the individual student while corresponding to the difficulty level required by respective curricula. An important difference between this category and the appropriately challenging support in the category *Constructive and appropriately challenging support* was that in *Optimal challenge for student and regarding school requirements*, the students perceived the challenge level as appropriate when no support was required. In *Constructive and appropriately challenging support*, the students described that their teachers successfully identified on which challenge level they may settle their support when support was required.

Turning to the next category *Feedback and evaluation* (T4), *Task-focused, constructive feedback* (T4.1) was the most frequently mentioned reason as to why the students felt competent. This subcategory was characterized by positive and informative teacher feedback, and by meeting the teachers’ expectations. Within this category, the students often described that they felt competent because the teachers generally gave sufficient feedback, or specifically gave positive feedback (e.g., female student SM03, 14 years old, reported in interview segment 4 “[I think I might have felt competent in this situation because] maybe being praised by the teacher has given me sort of a push.”). Additionally, we subsumed grades under the inductive subcategory *Feedback through grades or scores* (T4.2) since the students frequently reported them as helpful in order to feel competent. The students thereby frequently highlighted the informative character of grades (e.g., female student KM01, 15 years old, stated in interview segment 34 “[This gave me another confirmation that I had, uh, written a good test]. Because there was also a grade underneath, yes.”). Surprisingly, teacher feedback specifically oriented toward the intraindividual reference norm orientation (*Improvement-focused, constructive feedback and evaluation* [T4.3]) seemed less relevant for the interviewed students’ competence satisfaction. It was only mentioned once and defined by teacher feedback that highlights improvements and individual developments over time.

As expected, the students frequently described some key elements of teacher autonomy support: Referring to the category *Autonomy-supportive teaching* (T5), the most frequent subcategory was *Participation possibilities and autonomy-supportive interaction* (T5.1). In this subcategory, the students described that they felt competent because teachers provided opportunities to actively interact with the classroom environment (including teacher–student interactions), and because teachers engaged in an active, respectful, and interested dialogue with their students. For example, the students highlighted the importance of opportunities for every student to actively engage in class (e.g., male student AT02, 15 years old, explained in interview segment 12 “[I think I felt competent in that situation because the teacher felt you were capable of doing that] and gave you the chance.”), and respectful teacher-student-interactions (e.g., female student AV03, 15 years old, stated in interview segment 52 “[Teachers who help me feel competent] deal respectfully with the students.”).

Beyond that, the students frequently emphasized factors subsumed under the next categories *Classroom management* and *Opportunities for collaborative working and peer interaction* as reasons for their competence satisfaction in class. The category *Classroom management* (T6) described that teachers effectively organized the classroom environment in order to establish a high and productive time-on-task for the students. For example, the students mentioned that they felt competent when teachers created a quiet working atmosphere, e.g.,

[To make me feel competent teachers could] make sure that it is quiet in class [because sometimes I have the feeling that many teachers somehow struggle to be assertive so that many students in my class don’t care and don’t listen to them. Then, it’s difficult to keep them quiet]. (Female student AT04, 15 years old, in interview segment 44)

Furthermore, the students felt competent when teachers succeeded in maintaining rules and guidelines, e.g.,

[[Teachers who help me feel competent] do not, it sounds harsh now, abuse their power. Rather, they are relatively on an equal footing.] But nevertheless, that they are still able to take action, I would say. That’s pretty important to me. (Female student CM11, 15 years old, interview segment 53)

The category *Opportunities for collaborative working and peer interaction* (T7) included the promotion of teamwork opportunities and interaction among classmates in class. Here, the students frequently reported feeling competent when teachers created opportunities for peers to interact with each other, such as in small group working or exchange among seating partners. Moreover, the students often felt competent when teachers created opportunities for classroom conversations with the whole class, for instance, in class discussions (e.g., female student AT04, 15 years old, reported in interview segment 54 “Not simply working individually on any tasks but maybe having more of a class discussion [helps me feel competent in class again].”).

### Teacher factors and student–teacher-relationship factors

However, in addition to the teaching factors, the students described *Teacher factors and student-teacher-relationship factors* (TR; describing teachers’ person-related competencies, characteristics, traits, and attitudes that contributed to students’ competence satisfaction through the students’ perspectives) as relevant reasons for perceiving competence satisfaction in class. This inductive main category involved three categories (e.g., *Teacher personality, characteristics, and attitudes*). For instance, several students told the interviewers that they felt competent because teachers mastered their school subject well and evaluated students competently (i.e., *Teacher’s professional and diagnostic competence* [TR2]). A positive student-teacher-relationship (i.e., *Positive student-teacher-relationship* [TR3]) was also mentioned several times. It was characterized by the student’s positive attitude toward the teacher and by the student’s perception of a good relationship with the teacher.

However, among our participants, the most salient category in this main category was *Teacher personality, characteristics, and attitudes* (TR1). This category was characterized by any teacher characteristics, personality traits, attitudes, and understandings of the teacher role that did not describe specific teaching behaviors or competencies. For example, the students felt competent because the teachers were generally kind and affable. Additionally, the students frequently reported that they felt competent because of relaxed teachers who were not too strict (e.g., female student SD03, 14 years old, stated in interview segment 53 “[Teachers who help me feel competent are] not necessarily too strict.”).

### Student factors

Moreover, we inductively added the main category *Student factors* (S) into the category system. This main category described students’ own skills, characteristics, attributes and attitudes that contributed to their competence satisfaction. It included six categories (e.g., *Student motivation and engagement*), with one of those categories (*Current mastery experience*) being further divided into three subcategories (e.g., *Notion of a currently successful interaction with teaching or exam material*).

In the main category *Student factors*, two factors were salient for 100% of the participants of our study: *Student motivation and engagement* as well as *Notion of a currently successful interaction with teaching or exam material*. With regard to the category *Student motivation and engagement* (S1), the students stated that they traced their competence satisfaction to their own motivation, preparation, and engagement such as in class or at home (e.g., female student KM01, 15 years old, explained in interview segment 19 “[I believe the reason why I felt competent on my part was] above all, that I personally tried to prepare myself for it in advance.”). In the category *Notion of a currently successful interaction with teaching or exam material* (S2.1), the students described that they perceived competence satisfaction because they were successfully interacting with teachers, the teaching material, or with the exam material. For example, the students described that they felt competent because they understood the learning material, were capable of doing something, or recognized that they had done or understood something correctly (e.g., female student KM01, 15 years old, commented in interview segment 4 “I realized that what I did was right.”).

In contrast to the related *Teaching factors* subcategory *Improvement-focused, constructive feedback and evaluation*, more than 50% of our sample also considered factors describing the *Notion of own learning improvement* (S2.2) as a reason for their competence satisfaction in class. This subcategory was characterized by descriptions in which the students compared their current actions or achievement with their prior actions or achievement, and in which they recognized learning improvements. For example, the students chose previous situations and the beginnings of current situations as benchmarks. They also explicitly addressed learning gains or improvements (e.g., female student SD03, 14 years old, reported in interview segment 8 “As opposed to back in the day, I improved.”).

Turning to the two last *Student factors* categories which were relevant for more than 50% of our participants, the two categories *Successful emotional coping* and *Generalized self-perceptions of competence and control beliefs* both described factors that went beyond one single situation, stressing the dynamics of classroom environments. In *Successful emotional coping* (S3), the students described reasons for the transition from a competence frustration to a brighter side of students’ competence satisfaction (i.e., the reduction of competence frustration or the beginnings of competence satisfaction) through specific thoughts, emotions or behaviors. For instance, the students reported that they felt competent because of putting situations behind them, positive thoughts, or relativizing thoughts, e.g.,

When I see that I got something right or that I was able to participate after all, I try not to let the bad drag me down. Instead, I try to focus on the bigger picture, for example, [to see] that I did better in another lesson, that it was just one lesson and I can still prove myself in the next lesson. (Female student SW12, 15 years old, in interview segment 42)

In the category *Generalized self-perceptions of competence and control beliefs* (S4), the students described that they felt competent because they were generally competent or had beneficial self-perceptions of competence (e.g., in a specific subject, topic, or task type) which went beyond one single situation in which the students felt competent. Additionally, this category included students’ general beliefs about being able to influence their own competence satisfaction or school outcomes. For example, the students stated that they felt competent because they generally felt that they were proficient or confident in a school subject or topic (e.g., female student DJ10, 15 years old, explained in interview segment 26 “[Because] I actually feel pretty confident in this subject.”).

### Peer climate and reciprocal peer support

Lastly, more than 80% of the interviewed students highlighted the importance of *Peer climate and reciprocal peer support* (P) as a factor that contributed to their competence satisfaction in class. This main category described a respectful, collaborative and learning-facilitating atmosphere among peers in which the students could or would help each other. For instance, the students reported that they felt competent because they were capable of helping other students (e.g., male student KA12, 15 years old, stated in interview segment 6 “Because I could help [others] with my skills.”). Also, the students felt competent because peers explained the learning material to them (e.g., female student SC06, 14 years old, reported in interview segment 66 “[To feel competent again] [Yes actually also, like, exchange with others] so that maybe not only the teacher, but also classmates explain things to you.”). An important characteristic of this main category was that the students traced the responsibility for these occurrences to interactions with their peers or to their peers but not to their teachers, or to the teachers’ teaching behaviors.

## Discussion

In this study, we attempted to widen researchers’ view on factors that contribute to students’ competence satisfaction at school by taking a qualitative, integrative, and student-oriented perspective. Specifically, we aimed to enrich and extend existing SDT-knowledge on which factors contribute to students’ competence satisfaction in realistic classroom settings. For this purpose, we combined a data-driven (explorative) and a theory-driven (descriptive) research design in which we integrated existing SDT assumptions as well as additional theoretical frameworks (i.e., classroom goal structure, perceived error climate, teaching quality, reference norm orientations; integrative approach). As one main finding of our qualitative content analysis approach, we identified and systematized 23 data- and theory-based factors (i.e., Teaching factors, Teacher factors and student–teacher-relationship factors, Student factors, Peer climate and reciprocal peer support) that contributed to students’ competence satisfaction in classroom contexts through the interviewed students’ perspectives. The most frequent categories were *Student motivation and engagement*, *Notion of a currently successful interaction with teacher, teaching or exam material*, *Clear communication and high-quality explanations*, as well as *Constructive and appropriately challenging support*. In contrast, the least frequent categories were *Improvement-focused, constructive feedback and evaluation*, *Opportunities to choose*, and *Positive student-teacher-relationship* (besides *Others*). Concluding, our data-based perspective first showed additional factors that seem to be beneficial for students’ competence satisfaction (e.g., student factors) through the interviewed students’ perspectives. Second, our theory-based perspective complemented quantitative SDT findings on need support and offered new conceptual insights into which teaching practices beyond the ones anchored in SDT (e.g., the fostering of a high mastery goal structure in class, teaching practices that characterize a high teaching quality) might facilitate students’ competence satisfaction at school through students’ perspectives (e.g., Rheinberg, 1980, 1983; Patall et al., 2008; Steuer et al., 2013; Lüftenegger et al., 2014, 2017; Dickhäuser et al., 2017; Praetorius et al., 2018; Vasconcellos et al., 2020). In the following, we present our specific findings along the sequence *Teaching factors, Teacher factors and student-teacher-relationship factors, Student factors*, and *Peer climate and reciprocal peer support*.

### Teaching factors contributing to students’ competence satisfaction

Expectedly, teaching factors were the most salient reasons for the interviewed students’ competence satisfaction (accounting for 48.70% of the relative category distribution). Based on SDT as our primary theoretical framework, we discuss our findings regarding the teaching factors along the need support variables structure, autonomy support, and relatedness support (Ryan and Deci, 2017). Moreover, we present additional factors we revealed within the teaching factors that could extend the need support literature in future research. The additional theoretical frameworks considered in our category system (i.e., achievement goal theory, perceived error climate, teaching quality, reference norm orientation theory) are used to extend existing knowledge on which teaching practices might be beneficial for students’ competence satisfaction through students’ views in the context of SDT.

#### Structure

In line with SDT, the categories *Clear communication and high-quality explanations*, *Constructive and appropriately challenging support*, as well as *Task-focused, constructive feedback* underpin the importance of structure for students’ competence satisfaction in class (Jang et al., 2010; Ryan and Deci, 2017, 2020; Aelterman et al., 2019; Vasconcellos et al., 2020). Together with the categories *Optimal challenge for student and regarding school requirements* as well as *Classroom management*, which are also discussable along the structure construct (Jang et al., 2010; Reeve, 2015; Ryan and Deci, 2017, 2020; Aelterman et al., 2019), these findings suggest that students might feel competent when teachers make expectations clear, give overviews, and provide appropriate help when necessary. Moreover, they emphasize the importance of positive and informative feedback, neither over- nor under-challenging tasks, and transparent as well as consistent rules. This description of competence-supportive teaching is in line with existing conceptualizations of structure in SDT (Jang et al., 2010; Aelterman et al., 2019; Ryan and Deci, 2020; Vansteenkiste et al., 2020).

Along with the benefits of qualitative approaches (Ryan and Deci, 2020; Vansteenkiste et al., 2020), we additionally revealed data-based factors by which the structure construct might be enriched in the educational setting. Whereas conceptualizations of clarifying structure have focused on overviews, transparent expectations, and the monitoring of students’ progress, our findings underlined the importance of understandable, precise, and sufficiently detailed explanations of the learning material for the students’ competence satisfaction (i.e., *Clear communication and high-quality explanations*; Aelterman et al., 2019). Moreover, our participants emphasized the indirect link of explanation quality and competence satisfaction via students’ understanding of the learning material, prompting future studies to consider both students’ motivational and cognitive functioning (e.g., Manganelli et al., 2019). This was particularly evident from the frequent consecutive occurrence of the categories *Clear communication and high-quality explanations* and *Notion of a currently successful interaction with teaching or exam material* across many participants in our study. Regarding the *Constructive and appropriately challenging support*, we conclude that an optimal challenge level might be important not only when providing tasks but also when providing help, which extends typical conceptualizations in SDT research (Jang et al., 2010; Guay et al., 2017; Aelterman et al., 2019; Ryan and Deci, 2020). Our findings also prompt future research to further investigate whether an appropriate challenge level should not only be defined regarding students’ current possibilities but additionally considering the challenge level of upcoming school requirements (e.g., final exams; Jang et al., 2010; Aelterman et al., 2019; Ryan and Deci, 2020).

To further enrich the structure construct, a theory-based and integrative perspective has been taken. The interviewed students mentioned several characteristics out of existing theoretical frameworks (e.g., teaching quality) as factors that contributed to their competence satisfaction in class (e.g., creating a quiet working atmosphere as an indicator for classroom management; Praetorius et al., 2018). These might extend typical conceptualizations in the need support literature. In line with existing research, our findings indicate that it is worthwhile to further investigate the link of mastery goal structure and students’ competence satisfaction (Kavussanu and Roberts, 1996; Cox and Williams, 2008; Quested and Duda, 2009; Halvari et al., 2011). They also prompt researchers to study whether the perceived error climate (Steuer et al., 2013), the teaching quality (Praetorius et al., 2018), and teachers’ reference norm orientations (Rheinberg, 1980, 1983; Dickhäuser et al., 2017; Lohbeck and Freund, 2021) might be related to students’ competence satisfaction in quantitative research. In order to facilitate the follow-up of our findings, Table 2 gives an overview about the theoretical frameworks and their dimensions that, based on our study, provide a fruitful foundation for further investigations of factors that can be beneficial for students’ competence satisfaction from a structure perspective. As can be seen in Table 2, the teaching factors derived from the different theories could be classified into common categories based on the interviewed students’ perspectives in the present research. These results are promising for future research, as this should facilitate to answer the call for integrative recommendations to practitioners that overcome conceptual overlaps between different motivational theories (Anderman, 2020).

**TABLE 2:** Dimensions describing the factors that contribute to students’ competence satisfaction from a structure perspective.

Category	Theoretical framework	Dimension
Clear communication and high-quality explanations	Mastery goal structure (TARGET framework)	Time
	Teaching quality	Classroom management
Constructive and appropriately challenging support	Mastery goal structure (TARGET framework)	Evaluation
	Perceived error climate	Error tolerance, irrelevance of errors for assessment, teacher support following errors, absence of negative teacher reactions to errors, taking the error risk, analysis of errors, functionality of errors for learning
	Teaching quality	Cognitive activation
	Teachers’ intraindividual reference norm orientation	Individualized instruction, moderate challenge level
Appropriate challenge for students and regarding school requirements	Mastery goal structure (TARGET framework)	Task
	Teaching quality	Cognitive activation, student support
	Teachers’ intraindividual reference norm orientation	Individualized instruction, moderate challenge level
Classroom management	Teaching quality	Classroom management
Task-focused, constructive feedback	Mastery goal structure (TARGET framework)	Recognition
	Teachers’ criteria-oriented reference norm orientation	

For an overview of the depicted theoretical frameworks and dimensions, see Rheinberg, 1980, 1983; Steuer et al., 2013; Dickhäuser et al., 2017; Lüftenegger et al., 2017; Praetorius et al., 2018; Lohbeck and Freund, 2021.

However, even though we retained the theory-based category *Improvement-focused, constructive feedback and evaluation* in our category system for transparency reasons and because it arose once in our sample, feedback focusing on students’ intraindividual improvement was not salient for students’ competence satisfaction in our study. At first sight, this stands in contrast to existing literature (Rheinberg, 1980, 1983; Reeve, 2015; Dickhäuser et al., 2017; Rheinberg and Krug, 2017; Ryan and Deci, 2017). However, several methodological (e.g., the high specificity of this category compared to the other feedback categories) and theoretical explanation approaches (e.g., small effect sizes of teachers’ intraindividual reference norm orientation; context specificities under which we conducted this study) might have caused this finding. For instance, teachers might rarely be oriented toward the intraindividual reference norm in the regular school system in Germany where this study has been conducted. Alternatively, the students might not have noticed the teachers’ efforts to focus on intraindividual improvement when they gave their feedback. Specifically, in line with some initial difficulties to differentiate between the categories *Task-focused, constructive feedback* as well as *Improvement-focused, constructive feedback and evaluation* in our study, several students might not have differentiated between constructive, task-focused, and improvement focused feedback in the present study. These possible explanations might have made it difficult to link teachers’ reference norm orientations to students’ competence satisfaction within the applied study design. From an SDT perspective, further research (accounting for context-specific influences, e.g., by intervention studies) is required in order to understand whether intraindividual comparison standards are related to students’ competence satisfaction in classroom contexts.

#### Autonomy support

In line with SDT, the students additionally mentioned that participation opportunities, respectful teacher-student interactions, and teachers who were responsive to students’ views, needs, and interests facilitated their competence satisfaction in class. This is in line with conceptualizations of attuning and participative autonomy support as well as with empirical SDT findings on individuals’ competence satisfaction (Patall et al., 2008, 2018; Jang et al., 2010; Ryan and Deci, 2017, 2020; Eckes et al., 2018; Aelterman et al., 2019).

However, matching some inconsistent findings in past research (Patall et al., 2014; Steingut et al., 2017; Vasconcellos et al., 2020), some autonomy support facets were more salient among the interviewed students (*Participation possibilities and autonomy-supportive interaction*) than others (*interestingness and relevance*, *opportunities to choose*). One possible explanation could be that opportunities for engaging in active interactions with classroom environments (i.e., *Participation possibilities and autonomy-supportive interaction)* might represent more proximal reasons for the students’ competence satisfaction compared to the sense of being self-determined causers of such active student-environment-interactions (e.g., *opportunities to choose*). Other explanation approaches might be that interestingness, relevance, and opportunities to choose are not salient teaching practices in Germany or that some students might have overlooked their teachers’ efforts to provide opportunities to choose or rationales. As one fruitful approach to understand how students perceive autonomy-supportive practices and their influence on students’ competence satisfaction while controlling for context specificities, one could manipulate specific autonomy support facets within qualitative intervention studies and explore students’ competence experiences (e.g., via open questions). Evidently, our findings should also be investigated considering several moderating and mediating processes (e.g., Patall et al., 2014; Steingut et al., 2017; Vasconcellos et al., 2020).

Furthermore, our findings revealed additional factors that might improve existing prediction results for students’ competence satisfaction in future studies. For instance, from a data-based perspective, the students felt competent when teachers gave equal opportunities to all students to participate in class. This approach extends past need support conceptualizations (Jang et al., 2010; Ryan and Deci, 2017, 2020; Aelterman et al., 2019), is in line with research on adaptive teaching (Corno, 2008), and prompts SDT researchers to complement existing competence satisfaction research by considering teaching equality beyond differentiated instruction (Deci, 2009; Roy et al., 2013; Guay et al., 2017).

In line with structure, autonomy support might additionally be refined based on our theory-based approach. Specifically, the interviewed students stressed factors that described mastery goal structure, teaching quality, and teachers’ intraindividual reference norm orientations as salient reasons for their competence satisfaction at school (Rheinberg, 1980, 1983; Dickhäuser et al., 2017; Lüftenegger et al., 2017; Praetorius et al., 2018). Therefore, our findings give first hints that existing autonomy support conceptualizations might be extended by dimensions out of those frameworks. In order to facilitate the transfer of our findings to quantitative research, Table 3 shows the theoretical frameworks and the specific dimensions that, following our findings, seem to be a fruitful approach in order to investigate which factors are positively linked to students’ competence satisfaction from an autonomy support perspective. As discussed for Table 2, the analyzed teaching factors derived from the different theories could be classified into common categories in the present research. This potential integrability might facilitate the derivation of recommendations for practitioners in future research (Anderman, 2020).

**TABLE 3:** Dimensions describing the factors that contribute to students’ competence satisfaction from an autonomy support perspective.

Category	Theoretical framework	Dimension
Participation possibilities and autonomy-supportive interaction	Teachers’ intraindividual reference norm orientation	Flexible, present-oriented teacher expectancies
	Teaching quality	Student support
	Mastery goal structure (TARGET framework)	Authority
Interestingness and relevance	Mastery goal structure (TARGET framework)	Task
Opportunities to choose	Mastery goal structure (TARGET framework)	Task
		Authority

For an overview of the depicted theoretical frameworks and dimensions, see Rheinberg, 1980, 1983; Dickhäuser et al., 2017; Lüftenegger et al., 2017; Praetorius et al., 2018.

#### Relatedness support

Within the categories *Constructive and appropriately challenging support, Participation possibilities and autonomy supportive interaction* as well as *Opportunities for collaborative working and peer interaction*, the students also viewed factors attributable to relatedness support as factors that contributed to their competence satisfaction which is in line with first hints in the literature (Vasconcellos et al., 2020). In line with existing relatedness support conceptualizations (e.g., opportunities for individualized teacher–student conversations; Reeve, 2015; Sparks et al., 2015, 2016), for instance, the students felt competent because their teachers had an approachable, helpful, and interactive teaching style. They also felt competent because teachers invited the students to interact with each other (e.g., by working in groups). Hence, our findings prompt future SDT research to focus on relatedness support as a potential predictor of students’ competence satisfaction.

Additionally, our findings provide first evidence that might broaden existing relatedness support conceptualizations. For instance, through the interviewed students’ perspectives, it seemed essential for students’ competence satisfaction that teachers and students met as equals, that students felt treated fairly by the teachers, and that the teachers were patient with students’ learning difficulties. For example, the students found it helpful when teachers actively provided voluntary opportunities for getting additional assistance (e.g., building small groups for whom teachers give additional assistance). This is partly in line with Reeve (2015) who proposed teacher patience to be an autonomy-supportive measure.

From our theory-based perspective, we conclude that existing SDT knowledge on relatedness support might be refined based on the depicted theoretical frameworks. Specifically, the categories *Opportunities for collaborative working and peer interaction*, *Participation possibilities and autonomy-supportive interaction*, and *Constructive and appropriately challenging support* revealed several teaching practices that have been elaborated in the TARGET framework (e.g., authority; Lüftenegger et al., 2017), in the student support and cognitive activation dimensions out of the teaching quality framework (e.g., discursive and co-constructive learning; Praetorius et al., 2018), in reference norm orientation theory (Rheinberg, 1980, 1983; Dickhäuser et al., 2017), and in perceived error climate research (Steuer et al., 2013). In view of the requirements for further research on relatedness support in SDT, it might be helpful for future research to integrate those factors into existing relatedness support conceptualizations (Reeve, 2015; Sparks et al., 2015, 2016). Future studies might also investigate whether our qualitative findings can be replicated in quantitative research. Attempting to facilitate the follow-up of our findings, Table 4 represents an overview of our findings concerning the theoretical frameworks and their dimensions that seem to contribute to students’ competence satisfaction from a relatedness support perspective. As discussed for Tables 2 and 3, the interviewed students’ perspectives additionally indicate that, based on the analyzed teaching factors, it might be fruitful as well as feasible for future research to elaborate integrative recommendations for practitioners with regard to students’ competence satisfaction (Anderman, 2020).

**TABLE 4:** Dimensions describing the factors that contribute to students’ competence satisfaction from a relatedness support perspective.

Category	Theoretical framework	Dimension
Participation possibilities and autonomy-supportive interaction	Teachers’ intraindividual reference norm orientation	Flexible, present-oriented teacher expectancies
	Teaching quality	Student support
	Mastery goal structure (TARGET framework)	Authority
Constructive and appropriately challenging support	Mastery goal structure (TARGET framework)	Evaluation
	Perceived error climate	Error tolerance, irrelevance of errors for assessment, teacher support following errors, absence of negative teacher reactions to errors, taking the error risk, analysis of errors, functionality of errors for learning
	Teaching quality	Cognitive activation, student support
	Teachers’ intraindividual reference norm orientation	Individualized instruction, moderate challenge level
Opportunities for collaborative working and peer interaction	Mastery goal structure (TARGET framework)	Grouping
	Teaching quality	Student support
		Cognitive activation

For an overview of the depicted theoretical frameworks and dimensions, see Rheinberg, 1980, 1983; Steuer et al., 2013; Dickhäuser et al., 2017; Lüftenegger et al., 2017; Praetorius et al., 2018.

However, in line with past SDT research (Ahn et al., 2019; Vasconcellos et al., 2020), our category system indicated overlaps between relatedness support and the other need support variables from the interviewed students’ perspective. For example, the category *Participation possibilities and autonomy-supportive interaction* might be discussed both from a relatedness support perspective and an autonomy support perspective. Moreover, the category *Constructive and appropriately challenging support* can be viewed from a relatedness support perspective as well as from a structure perspective. Therefore, our findings prompt future research to investigate the empirical separability of structure, autonomy support, and relatedness support in the context of students’ competence satisfaction.

### Additional factors contributing to students’ competence satisfaction

Extending past SDT research that mainly focused on teaching practices (e.g., Ryan and Deci, 2020; Vansteenkiste et al., 2020; Vasconcellos et al., 2020), our findings also suggest that, from the interviewed students’ perspectives, additional factors beyond the teaching practices (accounting for 51.30% of the relative category distribution in our study when including *Others*) might be considered in order to understand why students’ competence satisfaction subjectively arises in class. In the following, we discuss the most salient additional factors through the interviewed students’ perspectives.

#### Teacher factors and student–teacher-relationship factors

Based on the main category *Teacher factors and student–teacher-relationship factors*, teacher factors might be a fruitful approach in understanding students’ perspectives on why their competence satisfaction arises in class (accounting for 9.22% of the relative category distribution in our study). For example, within the category *Teacher personality, characteristics, and attitudes*, the students described teacher agreeableness (i.e., kindness) and teacher characteristics presumably interpretable as generalized autonomy and relatedness supportive, as well as non-controlling orientations (e.g., “generally being attentive to students’ needs”) as factors that contributed to their competence satisfaction. This is in line with teacher personality research within and beyond SDT (Ryan and Deci, 2017; Kim et al., 2018; Reeve et al., 2018). According to teaching quality research and SDT research on relatedness support, the students also frequently reported teacher humor, enthusiasm, and teacher motivation as reasons for their competence satisfaction in class (Kunter, 2013; Sparks et al., 2015, 2016; Baier et al., 2019; Shahid and Ghazal, 2019; Ahn et al., 2021).

However, some inconsistent findings on the links between teacher personality and students’ motivational functioning prompt future research to follow up on our findings (Kim et al., 2018; Reeve et al., 2018; Baier et al., 2019; Khalilzadeh and Khodi, 2021). Moreover, students may not have differentiated between teaching behaviors and teachers’ orientations in our study, urging researchers to interpret our findings with caution.

#### Student factors

Through the interviewed students’ perspectives, student factors seemed crucial in explaining why students’ competence satisfaction arises in class (accounting for 34.87% of the relative category distribution). For instance, the students viewed their own motivation and engagement as one of the most important preconditions for their competence satisfaction, suggesting that students’ competence satisfaction might be considered as a predictor and as an outcome of motivation and engagement in future research. This is in line with longitudinal studies in which students’ need fulfillment and competence satisfaction have predicted students’ motivation and engagement, as well as vice versa (Papaioannou et al., 2006; Reeve and Lee, 2014).

Based on the main category *Notion of a currently successful interaction with teacher, teaching or exam material*, we conclude that, from students’ views, it seems essential for students’ competence satisfaction that students subjectively notice their own competence in current situations. This is in line with the hierarchical model of intrinsic and extrinsic motivation and with studies in which students who performed above-average did not necessarily feel competent (Miserandino, 1996; Vallerand, 1997). However, the conceptual similarity of this category with students’ competence satisfaction might indicate that it revealed indicators rather than reasons for students’ competence satisfaction (White, 1959; Ryan and Deci, 2017, 2020). Although we rigorously defined our coding units (i.e., reasons for students’ competence satisfaction; Mayring, 2014), future studies should address the empirical separability of the factors described in this category from students’ competence satisfaction. By doing so, one might disentangle definitional and preceding aspects of students’ competence satisfaction in class.

#### Peer climate and reciprocal peer support

*Peer climate and the reciprocal peer support* were further additional factors that contributed to the students’ competence satisfaction in class (accounting for 6.05% of the relative category distribution in our study). For instance, the students perceived the capability to help others, getting help from peers, asking of questions, and exchanging of ideas, views, and information among peers as reasons for their competence satisfaction in class. In line with first hints in the literature (Steuer et al., 2013; Vasconcellos et al., 2020), our findings thus shed light on how peer climate and peer interactions might influence students’ competence satisfaction in classroom contexts. Due to scarce research on how peer factors and students’ motivational processes interact in class (Núñez and León, 2015), our findings widen researchers’ view on why students’ competence satisfaction arises in class. They prompt future studies to focus on peer factors and peer interactions.

### Limitations

Despite our promising findings, some limitations must be addressed. First, qualitative research is object to researcher biases (e.g., sampling effects, anticipations, unconscious biases; Morse, 2015). For instance, although the applied qualitative content analysis approach is a transparent, rigorous, and rule-oriented approach to analyze qualitative material (Mayring, 2014), the interpretative coding of interview material remains a subjective process which can lead to subjective bias (Morse, 2015). Second, an important validity concern in qualitative research is to aptly describe the investigated phenomenon (Morse, 2015). In the present study, we investigated the interviewed students’ subjective experiences with and generalized beliefs about the factors that contribute to their competence satisfaction in class. According to Mayring (2007b), we hence may speculate that students in similar schools, in a similar age, in similar school subjects, and in similar life circumstances may report similar reasons for their competence satisfaction in class. However, the generalizability of the identified factors remains to be investigated since qualitative content analysis does not make claims of generalizability (Krippendorff, 1989; Mayring, 2007b). Furthermore, potential threats to the accurate description of the investigated phenomenon have to be considered. For example, although we conceptualized our interviews along typical SDT definitions (White, 1959; Deci and Ryan, 2000; Ryan and Deci, 2017, 2020), the applied operationalization of students’ competence satisfaction might be confounded with other self-perceptions of competence. In order to verify whether we described the phenomenon of interest, future studies should test whether our qualitative findings are replicable, for instance, in quantitative studies with well-validated SDT questionnaires (e.g., Heissel et al., 2018). Third, conclusions on whether the identified factors can predict students’ competence satisfaction in addition to structure, autonomy support, and relatedness support have yet to be drawn. Last, even though our study offered important insights into students’ perspectives on which factors contribute to their competence satisfaction based on a limited and purposeful sample, it was beyond the scope of this study to control for context-specific influences. For instance, the students may not have had the possibility to feel competent because of specific teaching practices if their teachers did not implement these practices in class. Therefore, future studies should combine the gains of qualitative approaches such as in our study with research designs which allow causal inferences (e.g., mixed-methods intervention studies).

## Conclusion

Following existing calls for qualitative research and giving insights into students’ views, this interview study explored students’ perspectives on which factors contribute to their competence satisfaction in class (Ryan and Deci, 2020; Vansteenkiste et al., 2020). From an SDT point of view, we first conclude that teaching factors within and beyond SDT were beneficial for students’ competence satisfaction from the students’ perspectives. Second, additional factors going beyond students’ perceptions of teaching practices, such as students’ perceptions of student factors (e.g., students’ motivation and engagement), teacher and student–teacher-relationship factors (e.g., teacher kindness), and peer climate factors (e.g., helping each other), played essential roles for the development of students’ competence satisfaction at school from the students’ perspectives. From a cross-theoretical point of view, our study shows the benefits of taking a qualitative, hybrid (or: integrated), integrative, and student-oriented perspective. The results of this study do not only enrich existing need-supportive measures by our integrative approach. They might also give new directions for the depicted additional theoretical backgrounds (i.e., classroom goal structure, perceived error climate, teaching quality, reference norm orientations). That is, the frameworks used in our study might benefit from integrating need-supportive measures anchored in SDT to enrich existing conceptualizations, and improve existing prediction results for the outcomes relevant to these frameworks (e.g., students’ reactions to errors in the error climate research; Steuer et al., 2013). This study might additionally inspire future research to reduce gaps not only within theoretical backgrounds by considering additional theoretical backgrounds, as in our study. In line with Anderman (2020), it might also inspire researchers to clarify differences and commonalities between related theoretical frameworks.

## Data availability statement

The datasets presented in this article are not readily available because the participants were assured the raw data would remain confidential and would not be shared. This was necessary due to the sensitive nature of the questions asked in this study. Requests to access the datasets should be directed to NR, nreymond@uni-bielefeld.de.

## Ethics statement

The studies involving human participants were reviewed and approved by the Ethics Committee of Bielefeld University. Written informed consent to participate in this study was provided by the participants and, if necessary by legal law, by the participants’ legal guardian/next of kin.

## Author contributions

NR developed the idea and study design (conceptualization), provided the study materials (investigation), and performed the data curation, visualization, methodology, writing of the original draft, as well as the project administration. NR, NG, and MW supervised the data acquisition which was conducted by two student teachers in the course of their master’s theses. NR and RN analyzed the data. SF supervised the project and provided the resources. All authors contributed to the article and approved the submitted version.

## Footnotes

1 The final category system can be obtained from the corresponding author of this study.
