# Supplementary material for: Why students feel competent in the classroom: A qualitative content analysis of students’ views
Source: Front Psychol. 2022 Oct 13;13:928801. doi: 10.3389/fpsyg.2022.928801 (PMC9612881; doi:10.3389/fpsyg.2022.928801)
Supplement: Supplementary file 1 [file Data_Sheet_1.docx]

**Appendix**

**Appendix A**

**Interview Schedule**

## Introduction to the Interview Situation

Hello, my name is *. I’m studying to become a teacher and I’m writing my master’s thesis in biology didactics at the University of *. Thanks for letting me conduct this interview with you. The interview will take about 30 minutes. I’ve just explained the study and this text (shows the declaration of consent) to you. Do you have any further questions?

Okay. Before we start the interview, it is important for me to say that you are free to answer the questions freely from your gut. There is no right or wrong. It’s all about your personal opinion. You won’t be rated either, so you can say exactly what you think. Also, you can always ask questions, for example, if you don’t understand the question.

(During the interview I’ll take short notes to make sure that I don’t forget anything in the interview.) Before we start: Do you have any questions about the procedure? Okay.

## Icebreaker

1. What grade are you in?
2. What’s your favorite subject?

## Interview Part I – Definition and Conditions

First of all, I have a general question for you:

1. What is going on inside you when you can effect something or when you are good at something?

*(If the question is not understood correctly: What does it comprise for you? What is going on inside you then? What crosses your mind and how does it feel?)*

Okay, great. You’ve just described what it feels like to be good at something or to be able to effect something. We also call that “competence satisfaction” or “someone feels competent in doing something”. That is exactly our topic: We do research on students’ competence satisfaction. This is not about whether you can actually do something really well (e.g., getting a good grade or managing a task) but about as how competent you evaluate yourself and how competent you feel. That’s why we will now use the term “to feel competent”. Can you relate to the term “to feel competent”?

*[If difficulties in understanding occur: Okay, imagine you start with jogging, and you realize that you’ve run well the first few times. When you are clear about the fact that you have run quite well and also quite far the last few times, then, you probably also know that you are now pretty good at jogging. At this point, you probably feel competent to go jogging. This “competence satisfaction” is exactly what we are going to talk about.]*

## Positive situation.

In class, you probably encounter situations repeatedly in which you feel sometimes more and sometimes less competent. I would like to learn what, from your perspective, does characterize those situations. That’s why I’ll keep asking you to tell me about classroom situations in which you feel competence or little competence.

Can you remember a current classroom situation (from the ongoing school year) in which you felt particularly competent? Please tell me about this situation.

1. What kind of situation was it? (*Lesson, assessment situation, others* 🡪 *explore the situation in detail to ensure sufficient clarity*)
   1. What had just happened?
   2. What did you do (in that situation)?
   3. In which subject did it happen?
2. How did you feel in that situation? *(Inquiry: What did you feel in this situation?)*
3. What thoughts crossed your mind? *(If only feelings but no thoughts are reported, ask again explicitly for the student’s thoughts.)*
4. What do you think: Why did you feel so competent in that situation?
   1. What had happened right before the situation (what possibly made you feel so competent)?
   2. What on your part could have made you feel competent in this situation?
      1. Did you do something specific that triggered that in you?
      2. What thoughts might have made you feel particularly competent in this
          situation? *(Did you think something specific that triggered that in you?)*
      3. What did your teacher do / say?
      4. How were your classmates involved in the situation?
5. How did the situation end?
   1. What did you do then? / How did you behave?
   2. What did your teacher do then? *(If not already mentioned: Did your teacher talk to you in this situation?)*
   3. What did it do to you that *[placeholder: you / your teacher]* did that? *(What did it trigger in you that [placeholder: you / your teacher] did that?)*
6. What do you think: What was the reason for your success? / What do you think was the reason it worked out so well in this situation?

## Negative situation.

Can you also remember a current classroom situation in which you felt little competence? Please tell me about this situation.

1. What kind of situation was it? *(Lesson, assessment situation, others 🡪 explore the situation in detail to ensure sufficient clarity)*
   1. What had just happened?
   2. What did you do (in that situation)?
   3. In which subject did it happen?
2. How did you feel in that situation? *(Inquiry: What did you feel in that situation?)*
3. What thoughts crossed your mind? *(If only feelings but no thoughts are reported, ask again explicitly for the students’ thoughts.)*
4. What do you think: Why did you feel little competence in that situation?
   1. What had happened right before the situation (what possibly made you feel
       little competence)?
   2. What on your part could have made you feel little competence in this
       situation?
   3. Did you do something specific that triggered that in you?
   4. What thoughts might have made you feel little competence in this situation? *(Inquiry: Did you think something specific that triggered that in you?)*
   5. What did your teacher do / say?
   6. How were your classmates involved in the situation?
5. How did the situation end?
   1. What did you do then? / How did you behave?
   2. What did your teacher do then? *(If not already mentioned: Did your teacher
       talk to you in this situation?)*
   3. What did it do to you that [placeholder: you / your teacher] did that? *(What did
       it trigger in you that [placeholder: you / your teacher] did that?)*
6. What do you think: What was the reason why you had this sort of failure? / What do you think was the reason why it did not work out so well in this situation?

*(If enough time left: Repeat one positive situation, two negative situations, then again one
 positive situation)*

## Interview Part II – Conditions in General

*(Rather skip the second positive and second negative situation than interview part II. Do interview part II in any case!)*

Thank you for sharing this with me. Now I’m going to ask you a few more general questions. When you look back to different situations: In general, what helps you in class to feel competent or what is important for you in class so that you can feel competent?

1. What would the lessons have to be like for you to feel competent?
   1. What could your teachers do to make you feel competent?
   2. What could you do in class to make yourself feel competent?
2. *If not already addressed by the student:* If you are currently feeling little competence, what will help you in class to make you feel better?
   1. What would the lessons have to be like for you to feel competent again?
   2. What could your teacher do to make you feel competent again?
   3. What could you do in class to make yourself feel competent again?

*[If nothing comes up: Pick the negative situation(s) up again: If you think back to the situation(s) where you felt little competence: What could your teacher have done in this situation to make you feel more competent?]*

1. What are the characteristics of your teachers who help you feel competent? *(If difficulties in understanding occur: What are they like?) (Inquiry: What do they specifically do?)*
2. What are the characteristics of your teachers who do not help you so much in making you feel competent? (*If difficulties in understanding occur: What are they like?) (Inquiry: What do they specifically do?)*

*(If nothing specific comes up referring to the questions 6 to 9, inquire after a while, like: What do you mean exactly? What does is it look like then? In case of doubt, also ask for a description of situations).*

## Demographic Questionnaire

Finally, a few short questions about yourself:

1. How old are you? __
2. Which gender do you assign yourself to? (a) male, (b) female, (c) diverse
3. Do you know what educational qualifications your parents have?
   1. What is your father’s highest school-leaving qualification: (a) lower secondary
       school leaving certificate, (b) intermediate school-leaving certificate, (c)
       advanced technical college entrance qualification (or: advanced technical
       college entrance qualification), (d) general qualification for university
       entrance, (e) no graduation?
   2. What is your father’s highest professional qualification: (a) completed
       university degree, (b) completed apprenticeship, (c) others, namely: __?
   3. What is your mother’s highest school-leaving qualification: (a) lower
       secondary school leaving certificate, (b) intermediate school-leaving
       certificate, (c) advanced technical college entrance qualification (or: advanced
       technical college entrance qualification), (d) general qualification for
       university entrance, (e) no graduation?
   4. What is your mother’s highest professional qualification: (a) completed
       university degree, (b) completed apprenticeship, (c) others, namely: __?
4. What grade do you currently have in subject * *(situation 1)*? __
5. And what grade do you currently have in subject * *(situations 2, 3, and 4)*? __

Thank you for talking to me. That really helps me a lot. Now you can pick a candy. I hope there’s something good for you in there!?

Last but not least, I would like to generate a code with you. This enables us to assign your data to your person in case you or your parents want us to delete your data ahead of time. I’ll write this code for you on this letter which is for your legal guardians so that you can keep it safe at home. The code is made up of the following four characters:

1. First letter of your mother’s first name: __
2. First letter of your father’s first name: __
3. and your month of birth (if your month of birth only has one digit, please put a zero in front of it, e.g., March is equal to 03): __ __

**Appendix B**

**Transcription Rules**

The interviews were transcribed and anonymized based on Dresing and Pehl (2018), Selting et al. (2009), as well as on Kuckartz (2010, 2018). The transcription rules are presented in the following.

1. The interviews are transcribed verbatim.
2. Dialects are translated into standard German as verbatim as possible.
3. Punctuation is smoothed out for the sake of readability. When a brief lowering of the voice or an ambiguous emphasis occurs, a period is used rather than a comma. The meaning of the sentence remains unchanged.
4. The systematic distinctness, completeness and readability are adhered while transcribing the interviews.
5. Mentioned names and sites are anonymized (<name>).
6. Notation rules:
   1. Characters and abbreviations are written out (e.g., Percent, Meter).
   2. Abbreviations of words such as "runtergehen " instead of " heruntergehen" or "mal" instead of "einmal" are exactly written as they were spoken in the interview ([English example: ’til” instead of “until”]).
   3. English terms are handled according to the German spelling rules concerning upper and lower case letters.
   4. Second-person salutatory pronouns (in German: “du” and “ihr” [you]) are written in lower case. Polite pronouns (in German: “Sie” and “Ihnen” [you; no existing equivalent in English]) are capitalized.
   5. Numbers are presented as follows:
      1. Numbers zero through twelve are expressed as words within continuous text. Numbers higher than twelve are expressed as numerals within continuous text.
      2. Numbers with short names (especially round numbers) are expressed as words (e.g., twenty, one hundred, three thousand).
      3. Decimal numbers and mathematical equations are expressed as numerals (e.g., "3,5" and "4 + 5 = 9").
      4. Approximate numbers are expressed as words. Exact numbers are expressed as numerals (e.g., "The fifty million euros in state aid").
      5. Follow the conventions in order to present numbers, if available. For example, house numbers, page numbers, telephone numbers, account numbers, dates, and the like are expressed as numerals (e.g., "on page 11", "at the marketplace 3").
   6. Idioms are transcribed verbatim and in standard German (e.g. "übers Ohr hauen” instead of “über das Ohr hauen”; [English example: “getting outta hand” instead of “getting out of hand”]).
   7. If direct speech is quoted, place the direct speech in quotation marks (e.g., and then I said, "Well, let's see").
   8. Single letters are capitalized (e.g., “like bird with a B”).
   9. Lists are marked by a capital letter without parentheses.

**Table B1**

*Implementation Examples of Specific Transcription Rules During Transcription*

| Transcription rule | Implementation Examples |
| --- | --- |
| Breaks: The number of dots represents the number of  seconds | (.) (..) (…) |
| More than three seconds 🡪 estimated duration of speaking  break | (2min) |
| Quick, immediate connection to previously spoken | = |
| Strong accentuation | that? |
| Nonverbal, interview-related behaviour | *[shrugging shoulders]* |
| Incomprehensible passage; for longer passages add reason | (inc.); (inc., microphone noises) |
| Presumed wording | (rather) |
| Alternative presumed wording | (very/rather) |
| Word slurs are not transcribed, but approximated to   the written language | “I wanna see it” 🡪 “I want to see it” |
| The form of a sentence is retained, even if it contains syntactical errors | “I went at the shopping mall” |
| Aborted words or sentences (stuttering is smoothed out if it is not interview-related) | “I was think/ very worried” |
| Emotional nonverbal expressions. Indicate duration if possible | ((laughs)), ((exhales for 2 sec)) |
| Explanation of the meaning of the sentence / affirmating / negating | Mmmhm *(negating)* |
| Unrelated events | [phone rings] |
| Signals of delay | Uhm, ah |
| Change of speaker: interviewer & interviewee  Each speaking contribution receives its own paragraph. Place a free, empty line between speakers. Timestamps are at least inserted at the end of each paragraph | I: (interviewer) and  P: (interviewee) |
| Overlapping speaking: Use // at the beginning of the interjection. The text that is spoken at the same time is written within //. The interjection of the other person which is also marked with // begins in a separate line | // |

*Note.* Table content (Dresing & Pehl, 2018; Selting et al., 2009) has been translated from German into English. Examples have been changed and adapted to the English language as far as possible.

**Appendix C**

**Segmentation Rules**

Following Chi (1997), we analyzed the entire interviews and not just interview samples. Only the students’ verbal utterances but not the interviewers’ questions were divided into segments (= meaningful units). Each segment contained one proposition (i.e., one episode, one idea or one piece of information which is comprehensible by itself), ranging from one word to one paragraph.

In order to determine the segments, we applied the following principles:

1. The segment’s specifity fits the research question’s specifity (i.e., the research question must be answerable given the grain size of the segments; Chi, 1997). Segmentation must be done in such a way that each complete segment can be assigned to exactly one category during coding.
2. An *inference* indicates the beginning of a new segment (Chi, 1997). However, the main decision criterion is whether a single piece of information also makes sense without the previous inference. In case of doubt, a coherent idea remains together. That is why a segment can contain up to one paragraph, as some subjects need more than a sentence to express an idea.
3. *Reasoning chains* which include more than one proposition are divided into one segment per proposition.
4. *Linguistic markers* are used as segmentation guides. Following Schilling (2006), relationships marked by conjunction (e.g., “and”, “or”, “but, “because”) should generally be dissolved to break down the statements into their basic parts. However, the final decision is based on content-related criteria. We only separate parts of sentences if the connection marked by linguistic markers is not necessary for the understanding of the sentence, and if the idea does not depend on this connection.
5. If a segment clearly separable from the surrounding segments is difficult to understand (e.g., due to a missing reference word; due to colloquial language), we insert the missing linguistic information in curly brackets.
6. Whereas duplicate words or repetitive sub-sentences are removed, embellishing or inefficient text components are largely left in the text, ensuring that the content remains unchanged.
7. Formally, the end of a segment is marked with “>>” and a new line.
8. The segmentation results are checked by a second member of our research team.

Referring to the specific research question of this manuscript, the units of analysis were defined in the following way:

1. The coding unit is one word, and minimally contains one proposition (i.e., one episode, one idea or one piece of information which is comprehensible by itself) describing one factor contributing to students’ perceived competence.
2. The context unit is one paragraph, and maximally contains one proposition describing one factor contributing to students’ perceived competence (Schilling, 2006; Tesch, 2013).
3. The unit of classification are all coding units out of one interview referring to factors contributing to students’ perceived competence since we chose a cross-interview approach (Mayring, 2014; Schilling, 2006).

References

Chi, M. T. (1997). Quantifying Qualitative Analyses of Verbal Data: A Practical Guide. *Journal of the Learning Sciences*, *6*(3), 271–315. https://doi.org/10.1207/s15327809jls0603_1

Dresing, T., & Pehl, T. (2018). *Praxisbuch Interview, Transkription & Analyse, Audiotranskription: Anleitungen und Regelsysteme für qualitativ Forschende* (8th ed.). dr. dresing & pehl GmbH. https://www.audiotranskription.de/wp-content/uploads/2020/11/Praxisbuch_08_01_web.pdf

Kuckartz, U. (2010). *Einführung in die computergestützte Analyse qualitativer Daten* (3., aktualisierte Aufl.). *Lehrbuch*. VS, Verl. für Sozialwiss. https://doi.org/10.1007/978-3-531-92126-6

Kuckartz, U. (2018). *Qualitative Inhaltsanalyse. Methoden, Praxis, Computerunterstützung* (4. Auflage). *Grundlagentexte Methoden*. Beltz Juventa. http://ebooks.ciando.com/book/index.cfm?bok_id/2513416

Mayring, P. (2014). *Qualitative content analysis: Theoretical foundation, basic procedures and software solution*. Beltz. http://nbn-resolving.de/urn:nbn:de:0168-ssoar-395173

Schilling, J. (2006). On the pragmatics of qualitative assessment: Designing the process for content analysis. *European Journal of Psychological Assessment*, *22*(1), 28–37. https://doi.org/10.1027/1015-5759.22.1.28

Selting, M., Auer, P., Barth-Weingarten, D., Bergmann, J. R., Bergmann, P., Birkner, K., Couper-Kuhlen, E., Deppermann, A., Gilles, P., Günthner, S., Hartung, M., Kern, F., Mertzlufft, C., Meyer, C., Morek, M., Oberzaucher, F., Peters, J., Quasthoff, U., Schütte, W., . . . Uhmann, S. (2009). Gesprächsanalytisches Transkriptionssystem 2 (GAT 2). *Gesprächsforschung : Online-Zeitschrift zur verbalen Interaktion*. https://orbilu.uni.lu/handle/10993/4358

Tesch, R. (2013). *Qualitative Types*. Taylor and Francis. http://search.ebscohost.com/login.aspx?direct=true&scope=site&db=nlebk&db=nlabk&AN=818508
